# Supplementary material for: Vestibular Schwannoma Volume and Tumor Growth Correlates with Macrophage Marker Expression
Source: Cancers (Basel). 2022 Sep 12;14(18):4429. doi: 10.3390/cancers14184429 (PMC9496830; doi:10.3390/cancers14184429)
Supplement: Supplementary file 1 [file cancers-14-04429-s001.zip › Supplement.pdf]

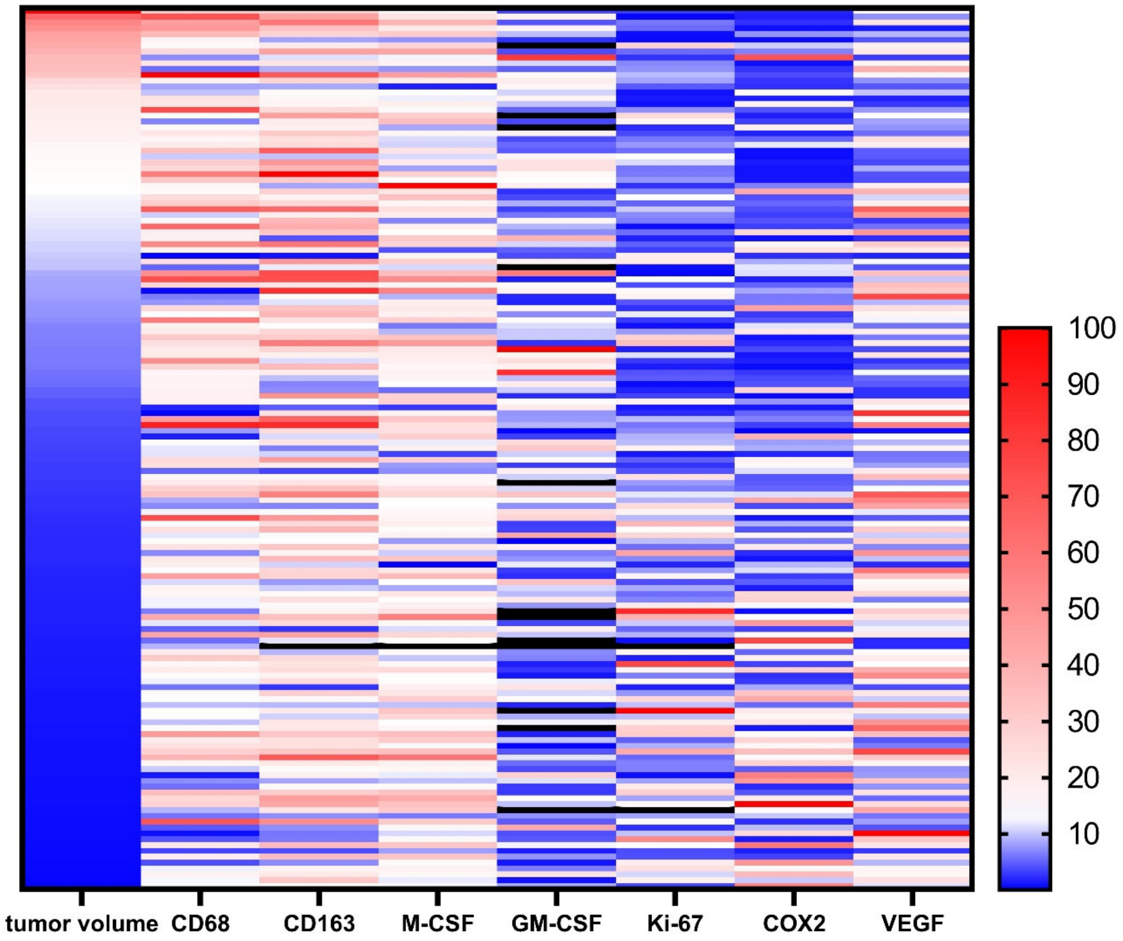

Figure S1. Heat map of mRNA data from 151 vestibular schwannoma tumors. mRNA was isolated from tumor samples and transcribed into cDNA as described in the materials and methods section. qPCR was performed using target gene specific primer (Table 1). mRNA expression of marker genes was normalized to GAPDH expression. The tumor volume was determined using the Brainlab software. The heat map shows the percentage of expression and tumor volume ordered by decreasing tumor volume of the 151 patients. Red marks a high marker expression/ large tumor volume and blue shows low marker expression/ small tumor volume.

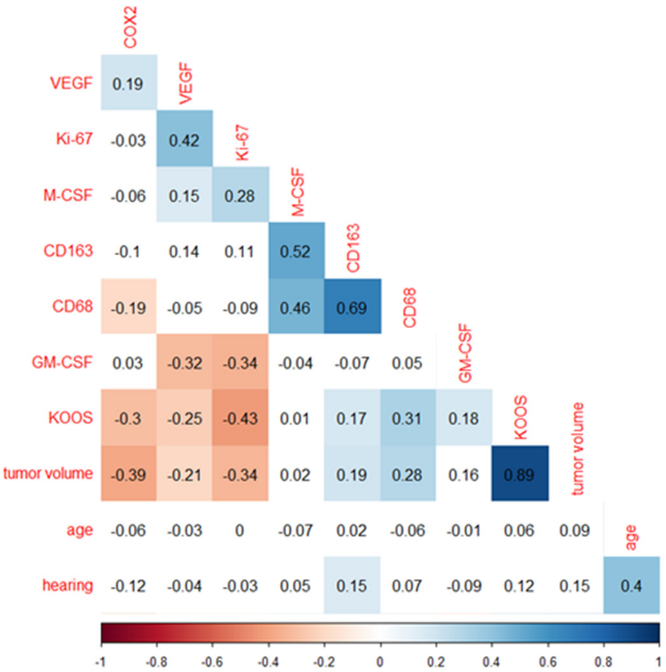

Figure S2. Correlation analysis with Spearman's rank correlation coefficient including Koos grade. Plot of correlations includes markers investigated, Koos grade, age and hearing class in 173 patients, correlation analysis of markers investigated and tumor volume in 151 patients. The numbers shown correspond to the correlation value r, also reflected by the color shading. Non-significant correlations (p≥0.05) are shown with a white background. Koos classification: grade 1 = small intracanalicular tumor, grade 2 = small tumor with protrusion into the cerebellopontine angle; no contact with the brainstem, grade 3 = tumor occupying the cerebellopontine angle with no brainstem displacement, grade 4 = large tumor with brainstem and cranial nerve displacement [19].

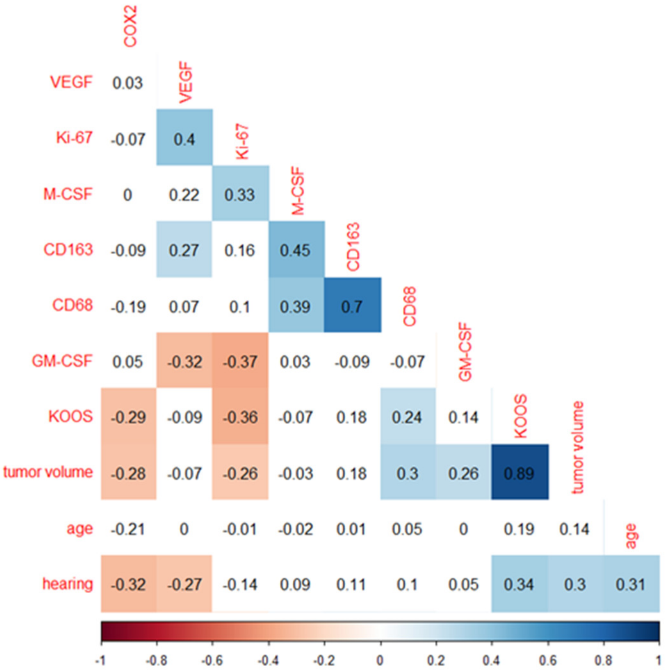

Figure S3. Correlation analysis in 74 patients with growth rate and Koos grade. Plot of correlations includes markers investigated, tumor volume, growth rate, Koos grade, age and hearing class with Spearman's rank correlation coefficient. The numbers shown correspond to the correlation value r, also reflected by the color shading. Non-significant correlations (p≥0.05) are shown with a white background. Koos classification: grade 1 = small intracanalicular tumor, grade 2 = small tumor with protrusion into the cerebellopontine angle; no contact with the brainstem, grade 3 = tumor occupying the cerebellopontine angle with no brainstem displacement, grade 4 = large tumor with brainstem and cranial nerve displacement [21].

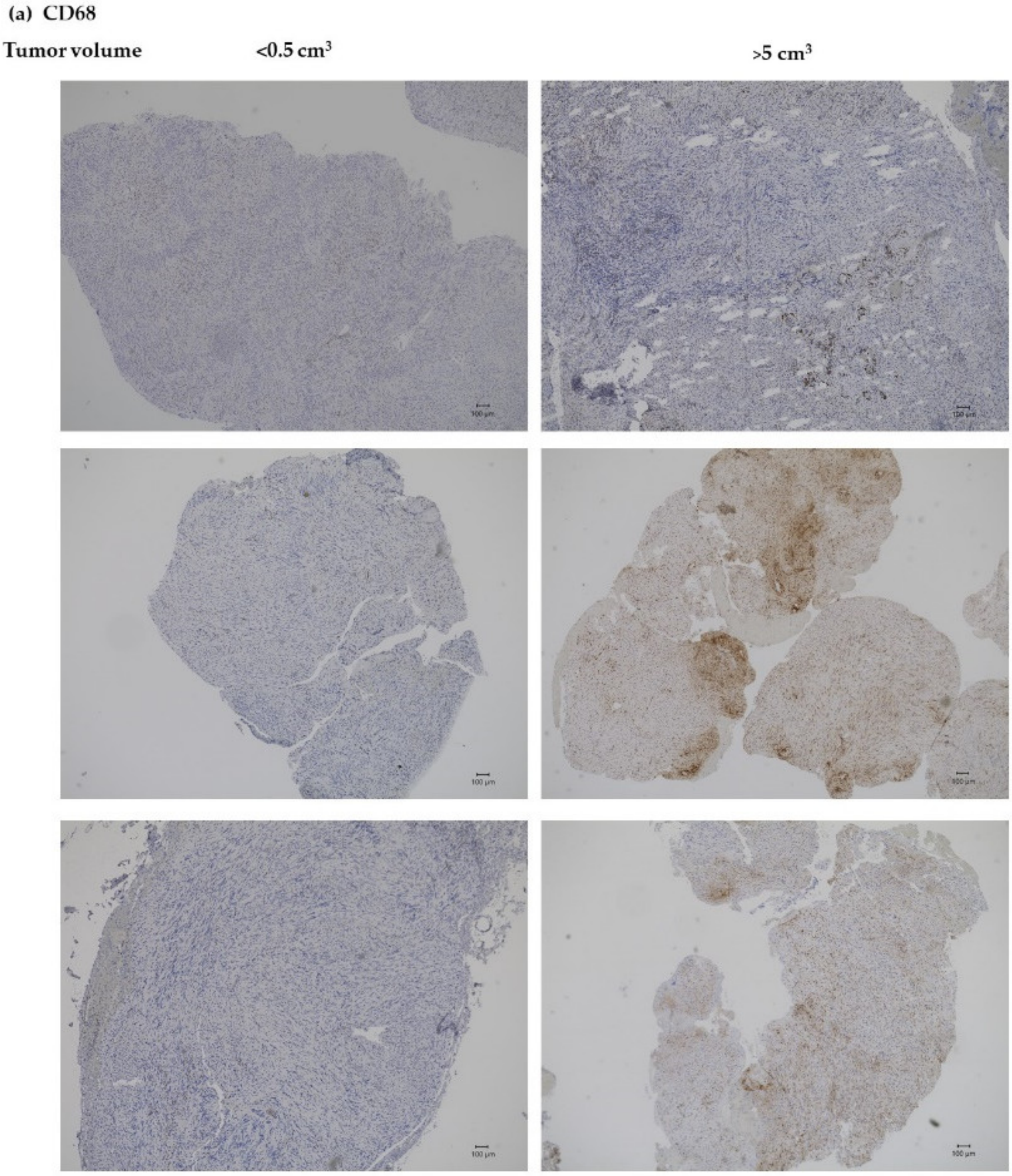

(b) CD163

Tumor volume <0.5 cm^3 >5 cm^3

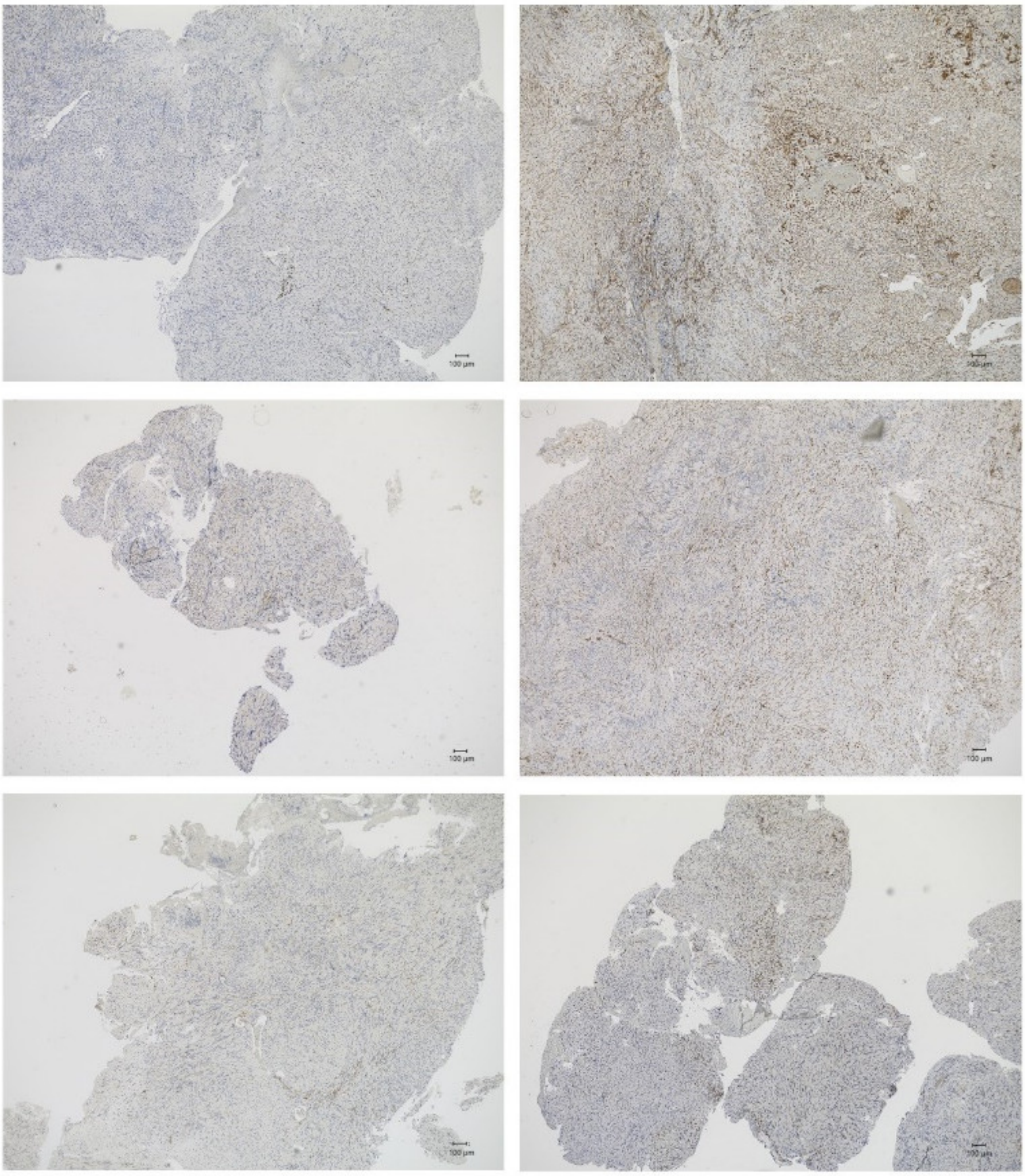

(c) Ki-67

Tumor volume **<0.5 cm^3** **>5 cm^3**

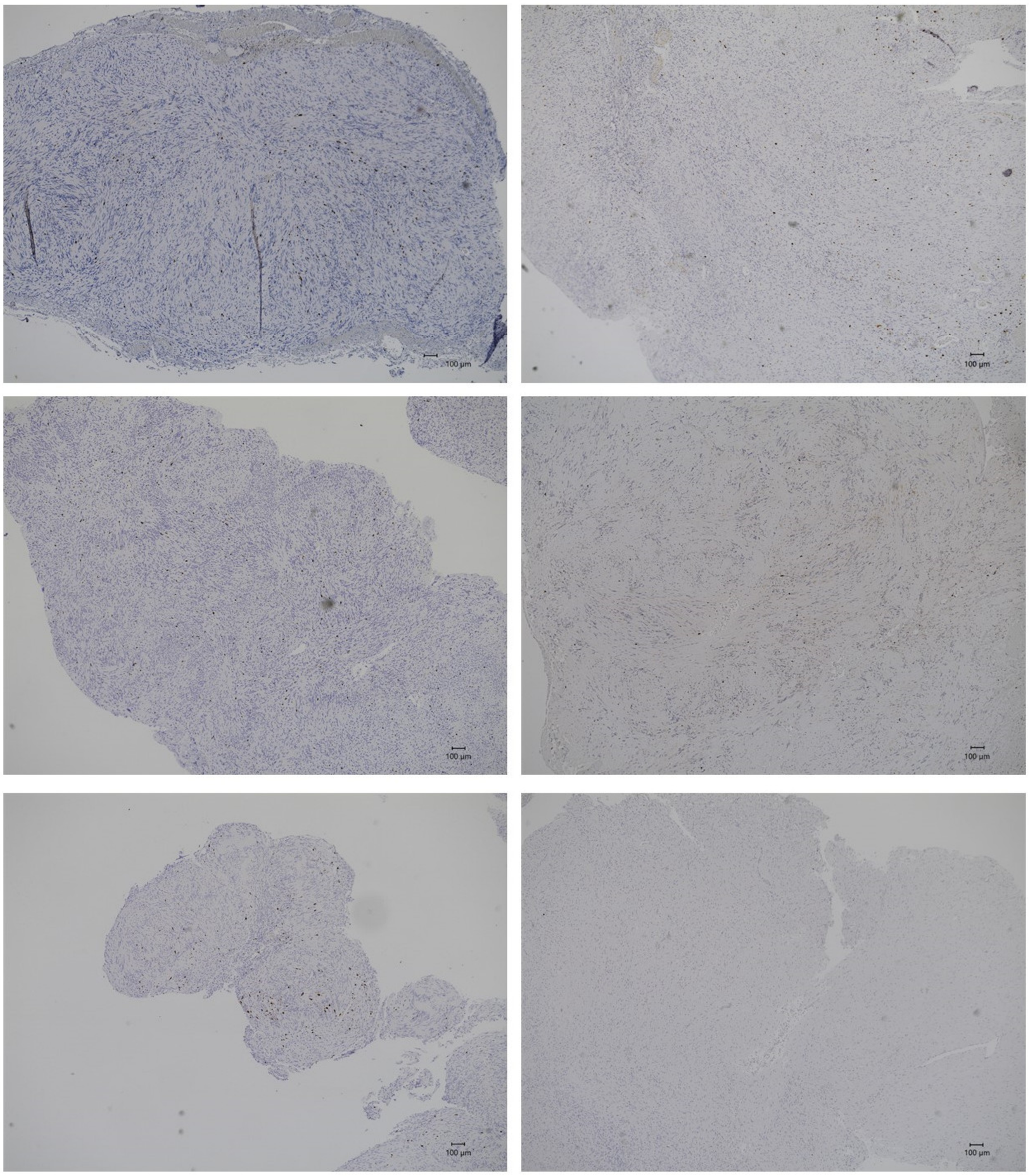

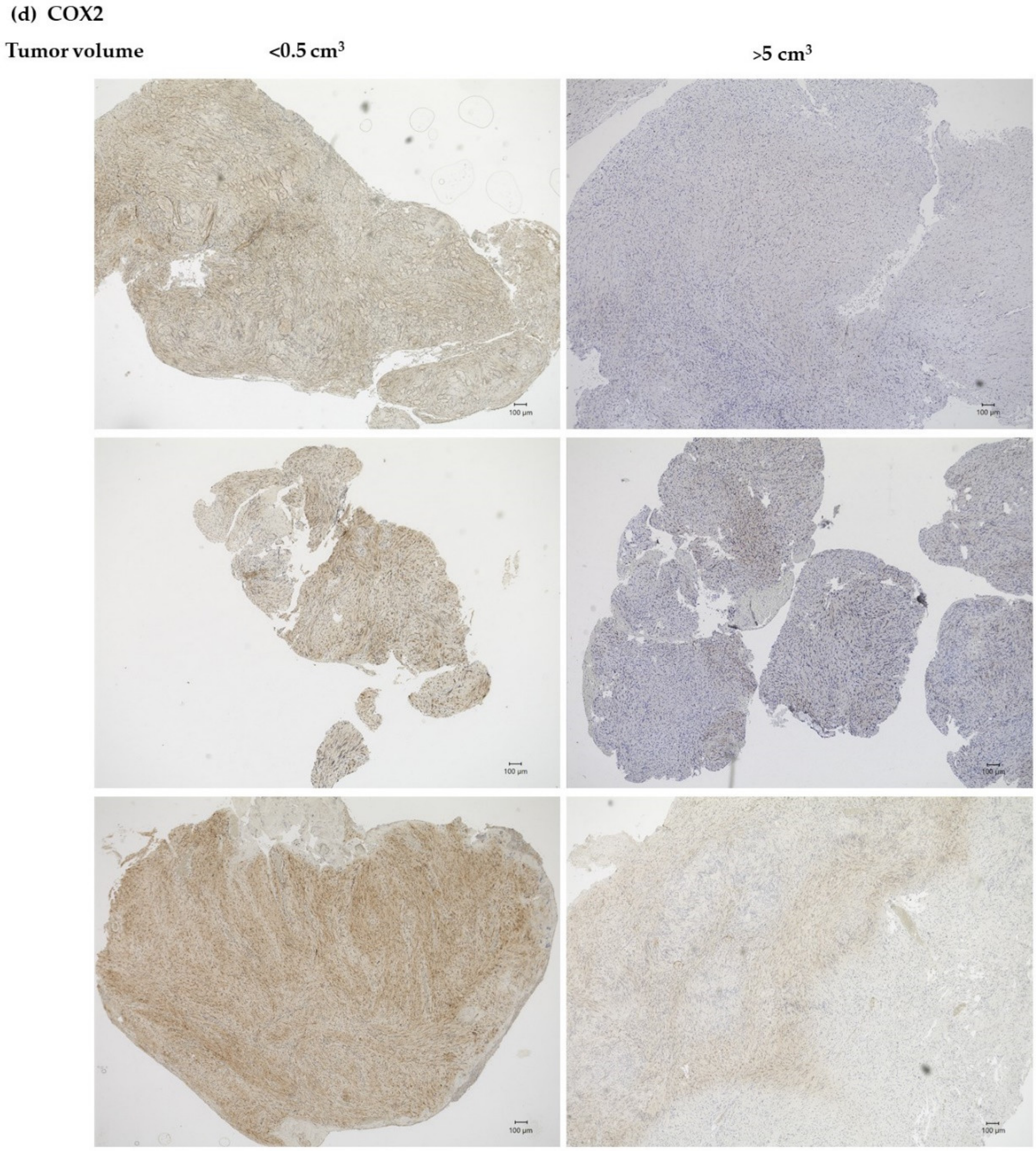

Figure S4. Overview images of IHC analyses of VS with different tumor volumes at 40x magnification. IHC analyses were performed as described in materials and methods and show staining with the specific antibodies against CD68 (a), CD163 (b), Ki-67 (c), and COX2 (d) from three randomly selected vestibular schwannoma samples with tumor volume <0.5 cm^3 (left column) and >5 cm^3 (right column).

(a) CD68

Tumor growth **<0.1 cm^3/year** **>1 cm^3/year**

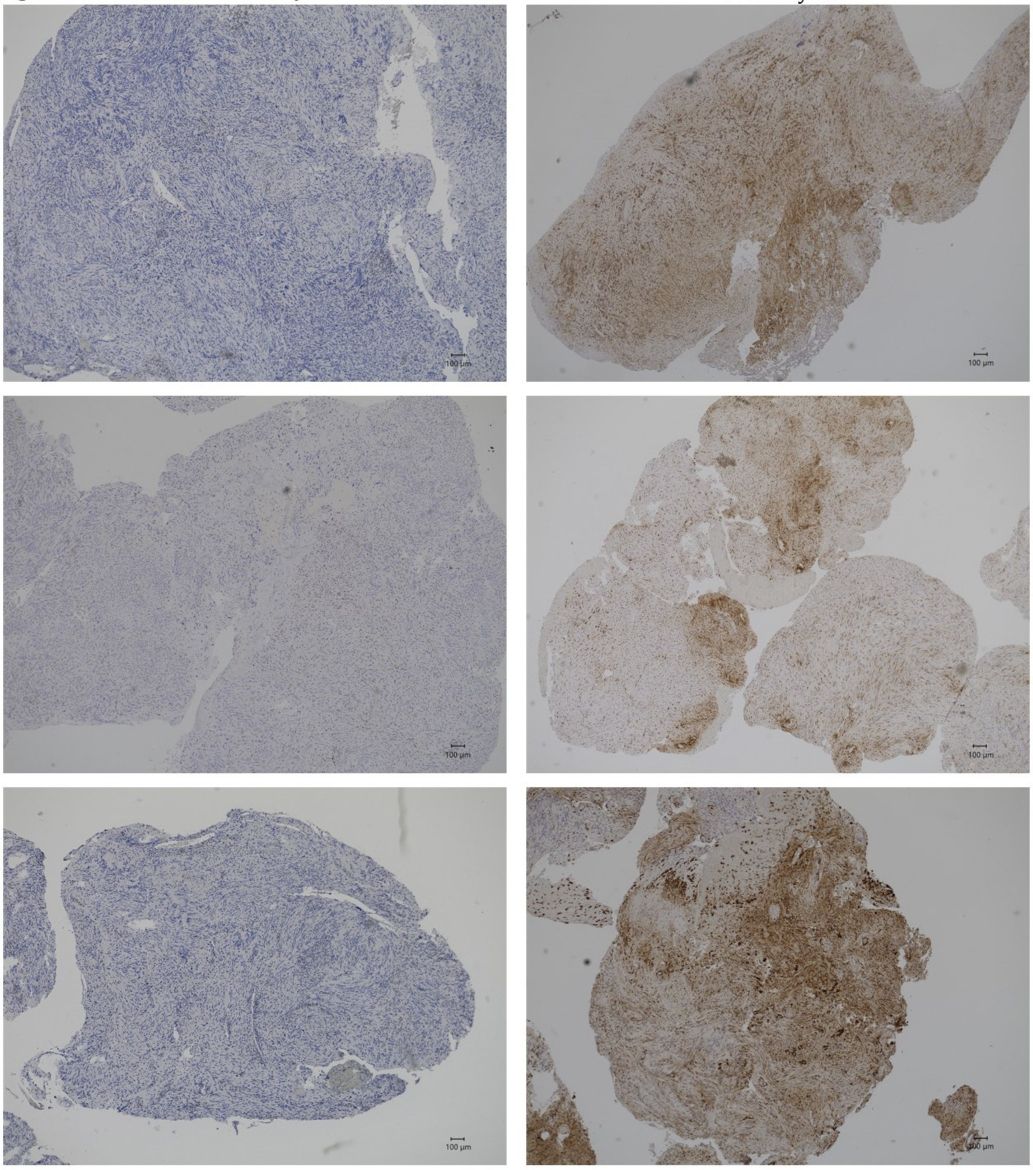

(b) COX2

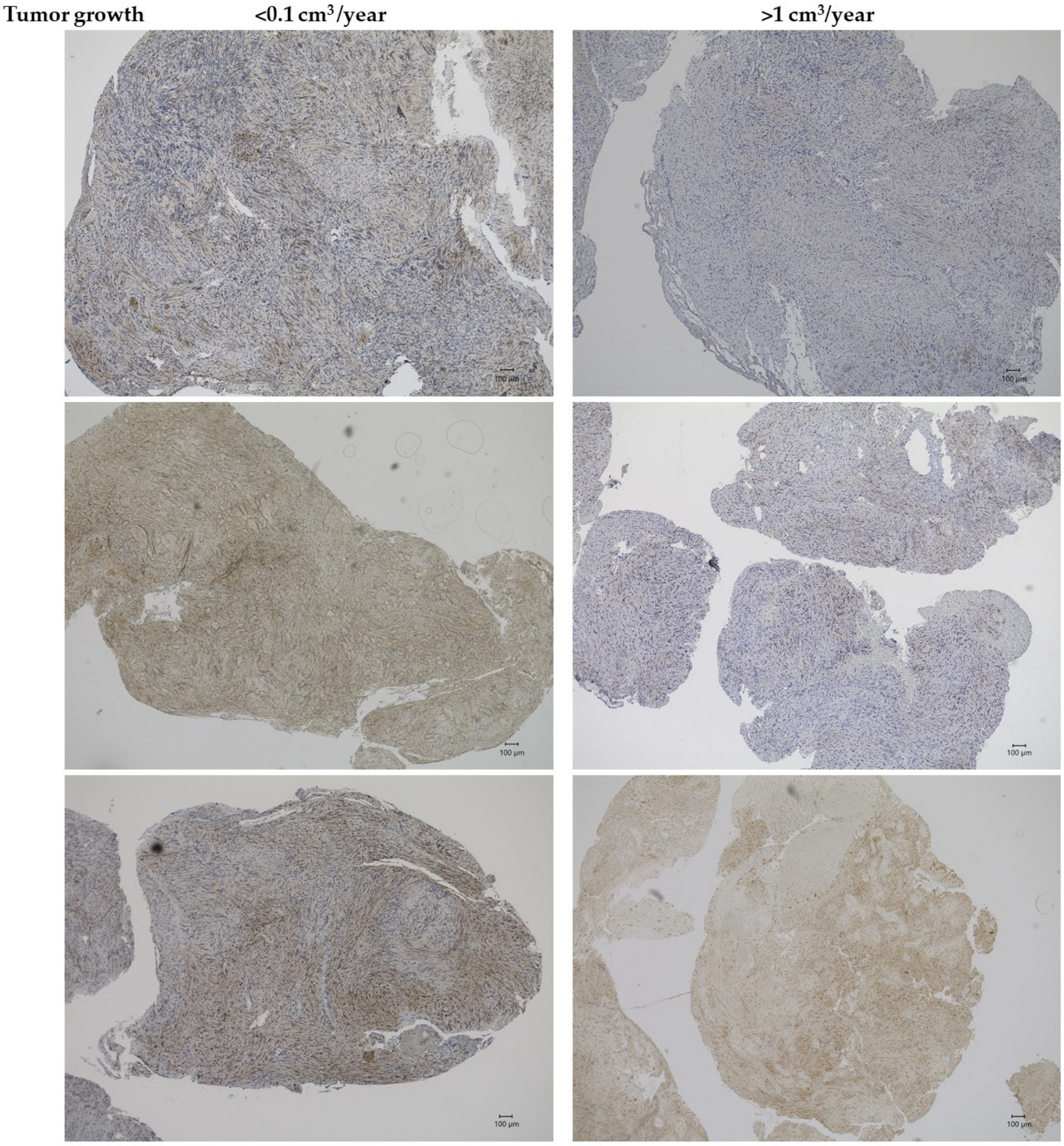

Figure S5. Overview images of the IHC analyses of slow and fast growing VS at 40x magnification. The microscope images show staining with the specific antibodies against CD68 (a) and COX2 (b) from three randomly selected vestibular schwannoma samples with tumor growth <0.1 cm^3/year (left column) and >1 cm^3/year (right column).
